# Quantification of epicardial fat volume using cardiovascular magnetic resonance imaging

**DOI:** 10.1186/1532-429X-16-S1-O112

**Published:** 2014-01-16

**Authors:** Tonye Teme, Bassel Sayegh, Mushabbar Syed, David Wilber, Lara Bakhos, Mark Rabbat

**Affiliations:** 1 Loyola University Medical Center, Maywood, Illinois, USA

## Background

A growing body of data has demonstrated a direct relationship between epicardial fat volume(EFV) and cardiovascular diseases. In fact, EFV has been shown to be an independent predictor of coronary atherosclerosis and atrial fibrillation. Currently, there is no standard protocol for assessing EFV using cardiac magnetic resonance imaging (CMR). Quantification of peri-ventricular EFV utilizing end diastolic short-axis cine sequences has been described; however, this technique is often challenging due to inadequate visualization of the pericardium. Oftentimes, the pericardium is better visualized during the systolic phase. Thus, we sought to determine the correlation and reproducibility of conventional EFV quantification using end diastolic with end systolic short-axis slices.

## Methods

**Figure 1:**
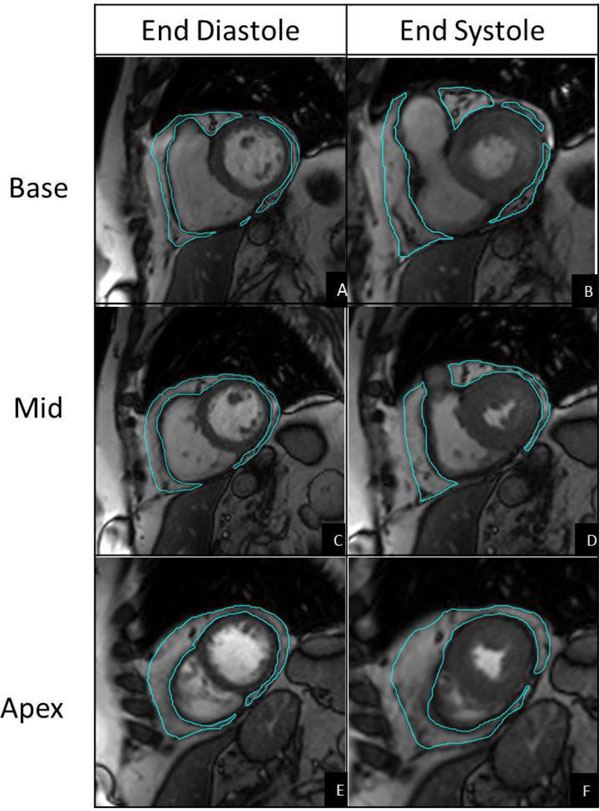
**Quantifying epicardial fat volume utilizing CMR**. Manual tracings were performed measuring the fat between the myo-epicardial border and viscero-parietal pericardial border in both end-diastole (A, C, E) and end-systole (B, D ,F).

We prospectively studied 20 patients with atrial fibrillation (AF) from November 1, 2010 to July 31, 2012 prior to AF ablation who underwent CMR. CMR images were acquired on a 3T scanner (Siemens Trio) by SSFP using a standard short axis stack through the ventricles. Epicardial fat was assessed volumetrically in consecutive short-axis views using the multi-slice method (MSM) during both end-systole and end-diastole (Figure 1). EFV measurements were compared using intra-class correlation coefficients (ICC), Pearson's correlation and Bland-Altman plots. The reproducibility of both EFV techniques was assessed by an additional investigator who was blinded to the results.

## Results

Mean EFV using end-systole were similar to end-diastole (79.8 +/- 45.74 ml vs 78.1 +/- 45.28, p = 0.91). End-diastolic epicardial fat volume (EDFV) slightly underestimates end-systolic epicardial fat volume (ESFV) by 2% (Table 1). Absolute difference between EDFV and ESFV was -1.7 ± 6.0 ml. ESFV demonstrates strong correlation and level of agreement with EDFV (Pearson Correlation = 0.99, ICC = 0.99). Interobserver variability (kappa) for EDFV was 0.82 (95% CI: 0.74-0.90) and 0.81 (95% CI: 0.74-0.88) for ESFV.

**Table 1:** 

Epicardial Fat Volume	Absolute Difference	Percent Difference	ICC	Pearson Correlation	R2
EDFV vs. ESFV	-1.7 ± 6.0	-2.0 ± 9.7	0.99°	0.99*	0.98*

Epicardial fat volume measurements are in mL, Values are presented as means and SD. EDFV = End Diastolic Epicardial Fat Volume. ESFV = End Systolic Epicardial Fat Volume. ICC = intraclass correlation. R2 = coefficient of correlation. ° 95% Confidence Interval = 0.98 to 0.99, * = p-value < 0.05.

## Conclusions

ESFV demonstrates strong correlation, level of agreement and reproducibility with EDFV. When the pericardium is inadequately visualized during the end-diastolic phase, ESFV derived from standard cine sequences offers an alternate method for evaluating epicardial fat with CMR.

## Funding

None.

